# Changes in Tumor Stem Cell Markers and Epithelial-Mesenchymal Transition Markers in Nonluminal Breast Cancer after Neoadjuvant Chemotherapy and Their Correlation with Contrast-Enhanced Ultrasound

**DOI:** 10.1155/2020/3869538

**Published:** 2020-11-17

**Authors:** Xiaoling Leng, Guofu Huang, Lianhua Zhang, Jianbing Ding, Fucheng Ma

**Affiliations:** ^1^Department of Ultrasound, The Affiliated Tumor Hospital of Xinjiang Medical University, Urumqi, 830011 Xinjiang, China; ^2^Department of Hematology and Oncology, The Fifth Affiliated Hospital of Xinjiang Medical University, Urumqi, 830011 Xinjiang, China; ^3^Basic Medical College, Xinjiang Medical University, Urumqi, 830011 Xinjiang, China

## Abstract

Nonluminal breast cancer has high early metastasis and treatment resistance, and neoadjuvant chemotherapy (NAC) is needed. The presence of cancer stem cells (CSC) and epithelial-mesenchymal transition (EMT) leads to poor prognosis. This study investigated the changes in CSC markers and EMT markers after NAC in nonluminal breast cancer and their correlation with contrast-enhanced ultrasound (CEUS) features and chemotherapy efficacy. Before NAC, the range of nonluminal breast cancer on CEUS was larger than that of two-dimensional ultrasound, but after NAC, it was significantly smaller than that of two-dimensional ultrasound and closer to the postoperative pathological size. After NAC, the enlarged lesions and perfusion defects were significantly less than those before NAC. The time-intensity curve showed the characteristics of slow-in, low enhancement, and low perfusion. Nonluminal breast cancer downregulated the expression of CSC markers and EMT markers after NAC, but the epithelial phenotype of nonluminal breast cancer with good response to chemotherapy was upregulated. In nonluminal breast cancer with poor response to chemotherapy, markers of CSC and EMT were highly expressed before chemotherapy. In conclusion, CEUS is better than conventional ultrasound in estimating NAC efficacy in this mode. CEUS can also predict the prognosis of nonluminal breast cancer before NAC with the characteristics of enhanced enlargement and perfusion defects. The contrast-enhanced time-intensity curve of lesions with relatively poor blood supply may have more CSC and EMT characteristics.

## 1. Introduction

Nonluminal breast cancer, which lacks hormone receptor, accounts for 25% to 30% of breast cancers and has different clinicopathological characteristics, treatment response, and prognosis, showing increased early metastasis ability and treat resistance [1]. Currently, most patients with nonluminal breast cancer receive neoadjuvant chemotherapy (NAC) [2]. NAC can lower the grades and stages of nonluminal breast cancer and eventually achieve breast conservation [3]. However, NAC fails in many patients due to acquired resistance, which may be caused by cancer stem cells (CSCs) [4]. CSCs have the characteristics of self-renewal and multidirectional differentiation potential and are closely related to tumorigenesis, growth, metastasis, and drug resistance [3]. The expression of CSC markers in nonluminal breast cancer is higher than that of luminal breast cancer, indicating higher numbers of CSCs in nonluminal breast cancer [5]. Epithelial-mesenchymal transition (EMT) is closely related to CSCs. In EMT, epithelial cells lose intercellular tight connections and polarity and acquire mesenchymal cell characteristics [6]. The occurrence of EMT in CSCs will promote the proliferation and migration of fibroblasts and induce angiogenesis, which also supports the association between CSCs and microvessels in breast cancer [7].

At present, there is controversy as to whether tumor size can be used as an evaluation index to predict the efficacy of breast cancer after NAC [8]. There are some limitations of response evaluation criteria in solid tumor (RECIST) guidelines. For example, changes in tumor morphology size may lag behind functional changes (tumor neovascular morphology or blood perfusion) [9]. The necrosis or fibrous scars in residual tumors after chemotherapy are hard to be distinguished on imaging [10]. Antiangiogenic drugs act on vascular endothelial cells and inhibit tumor microvessel formation, but do not cause significant changes in tumor size [11]. Using tumor size alone can overestimate or underestimate the treatment response [12]. Tumor blood flow perfusion belongs to the category of tumor functional imaging, and changes in blood flow perfusion after NAC in breast cancer are important for efficacy evaluation [13]. Contrast-enhanced ultrasound (CEUS) based on microcirculation perfusion can not only show tumor blood vessels but can also provide more information about microcirculation hemodynamic changes [14]. Ultrasound contrast agent is a true blood pool distribution contrast agent and is not affected by the peripheral matrix. Enhanced magnetic resonance imaging (MRI) can not only show tumors but also high signals in necrosis and inflammatory lesions after NAC treatment, thus leading to the overestimation of tumor size [15]. Therefore, CEUS may reflect the efficacy of microcirculation perfusion more accurately.

In our previous research, it was found that the extent and degree of enhancement, as well as the perfusion defect, correlated well with prognostic factors of breast cancer [16], indicating that, in addition to assessing the efficacy, CEUS can also assess the prognosis. However, the result of CEUS on prognosis is only preliminary. Herein, we analyzed CEUS parameters before and after NAC. The changes of CSC markers and EMT markers before and after NAC and their correlation with CEUS features were also analyzed. The value of CEUS in evaluating the prognosis of nonluminal breast cancer before NAC was discussed.

## 2. Materials and Methods

### 2.1. Subjects

This study enrolled 80 cases of nonluminal breast cancer patients from January 2017 to October 2018 at the Affiliated Tumor Hospital of Xinjiang Medical University. They were aged 24 to 78 years, with a median age of 51 years old. Inclusion criteria: (1) patients with previously untreated and pathologically confirmed primary stage II to III breast cancer; (2) pathology type: Her-2 overexpression and triple-negative breast cancer; (3) patients received surgical treatment. Exclusion criteria: (1) patients received surgery, chemotherapy, radiotherapy, or endocrine therapy before enrollment; (2) patients with luminal breast cancer of multicentric or bilateral; (3) patients received chemotherapy as palliative rescue treatment and did not receive surgery. For control, 50 patients with benign breast lesions were selected, including 20 cases of fibroadenoma, 20 cases of proliferative lesions, and 10 cases of intraductal papilloma. Written informed consent was obtained from each patient. This study was approved by the Affiliated Tumor Hospital of Xinjiang Medical University.

### 2.2. Chemotherapy and Efficacy Evaluation

Six cycles of TAC chemotherapy, including paclitaxel liposomes 135-175 mg/m^2^ or docetaxel 75 mg/m^2^, d1; pirarubicin 40 mg/m^2^, and d1; cyclophosphamide 600 mg/m^2^ intravenous drip, were performed. The treatment efficacy was evaluated according to Miller-Payne (MP) score after surgery. Level I: no change in the total number of cells; Level II: reduction of the number of tumor cells by 30%; Level III: reduction of the number of tumor cells by 30%-90%; Level IV: reduction of the number of tumor cells by 90%; Level V: all tumor cells disappeared, and only microscopic interstitial residues of blood vessels were found. Levels I-III were defined as nonmajor histological response (NMHR), and those of IV-V were considered major histological response (MHR).

### 2.3. CEUS and Conventional Ultrasound

The GE Logic E9 color Doppler ultrasound system with 9 L-4 probe was used. Patients were in a supine position. Two-dimensional ultrasound was performed to check the shape, size, and edge of the tumor. Then, the contrast agent was quickly injected into the elbow vein of the upper limb of the healthy side. After injection of contrast agent, dynamic images were immediately recorded for 2 min and 30 s until enhancement decrease. The qualitative indicators of CEUS were analyzed, including lesion range and perfusion defects. The time-intensity curve (TIC) was obtained. The peak intensity (PI), time to peak (TTP), arrival time (AT), and area under the curve (AUC) were calculated.

### 2.4. Sampling

Cancer tissues and paracancer tissues (1 cm away from cancer tissues) were collected by biopsy. All lesions were sampled before and after NAC. Before NAC, a thick needle biopsy was performed under the guidance of CEUS. Cancer tissues were collected from regions with rich blood perfusion, and paracancer tissues were collected at 1 cm from the cancer. After NAC, the tissues were collected from the same section using the same method.

### 2.5. Breast Tumor Cell Isolation

The collected tumor tissues were rinsed and cut to a size of 1 mm^3^. After rinsing and centrifugation, the samples were resuspended in culture medium. Collagenase (200 U/mL) and hyaluronidase (200 U/mL) were added for digestion at 37°C overnight. After centrifugation at 1000 rpm for 5 min, the supernatant was discarded. The cells in the precipitation were diluted to 1 × 10^6^/mL in serum-free medium, inoculated into culture flasks, and incubated at 37°C, 5% CO_2_. The medium was changed every 3 days to remove dead cells and necrotic tissues. The inverted microscope was used to observe the formation and growth of cells.

### 2.6. Flow Cytometry

The expression of CD44 and CD24 was analyzed by flow cytometry to identify CSCs. Briefly, 0.5 *μ*L of CD24-FITC (Thermo; 12-0247-42) or CD44-FITC (Thermo; 11-0441-82) was added to 100 *μ*L cells (1 × 10^6^/mL) and incubated at 4°C in the dark for 20 min. The cells were suspended with 500 *μ*L of cold PBS and transferred to a flow cytometer. The ratio of CD44+/CD24-/low was analyzed by BD FACSAria II (BD).

### 2.7. Real-Time PCR (qRT-PCR)

Total RNAs from breast tumor cells and tissues before and after NAC were extracted using Trizol reagent (ET111, TransGene). The RNA concentration was measured, and the integrity was checked by electrophoresis. cDNA was generated by reverse transcription, and qRT-PCR was carried out using TransScript One-Step gDNA Removal and cDNA Synthesis SuperMix (AT311, TransGene). The reaction contained 2 × SYBR Green Select Mix (5 *μ*L), Forward Primer (0.7 *μ*L), Reverse Primer (0.7 *μ*L), ROX (0.05 *μ*L), cDNA (1 *μ*L), and RNase-free water (up to 10 *μ*L). The primer sequences were shown in Table 1. The qRT-PCR reaction was 95°C, 2 min; and 40 cycles of 95°C, 5 sec, and 60°C, 30 sec. The relative mRNA levels were calculated using the 2^-*ΔΔ*ct^ method. hsa-Actin was used as the internal control.

### 2.8. Western Blot

Cancer tissues, paracancer tissues, and breast tumor cells were subjected to RIPA lysis. After centrifugation, total proteins were obtained. The protein concentration was determined by the BCA method. Then, the proteins were separated by SDS-PAGE and transferred to PVDF membrane (Millipore; Cat# IPVH00010). After blocking, the membrane was incubated with primary antibodies (E-cadherin (1 : 10000, ab40772, Abcam), *β*-catenin (1 : 5000, ab32572, Abcam), vimentin (1 : 1000, ab8978, Abcam), N-cadherin (1 : 5000, ab76011, Abcam), CD44 (1 : 2000, ab157107, Abcam), CD24 (1 : 200, MA5-11833, Invitrogen), and *β*-actin (1 : 800, D110001, Shanghai Health Worker) at 4°C overnight. After washing, the membranes were incubated with the secondary antibody at room temperature for 1 h. After washing, the signals were detected using Chemiluminescence Imaging System (ChemiScope Mini 3300; Shanghai Qinxiang Scientific Instrument Co., Ltd., Shanghai, China).

### 2.9. Immunohistochemistry

The tissue samples were fixed with formalin, embedded, and sectioned. The samples were then incubated with primary antibody and secondary antibody and finally developed by 3,3′-Diaminobenzidine. Five high-power fields were randomly observed in each slice, and 100 tumor cells were counted. The percentage of positive tumor cells were scored as follows: <5% (0 points), 5%~25% (1 point), 25%~50% (2 points), 50%~75% (3 points), and >75% (4 points).

### 2.10. Statistics Analysis

SPSS 21.0 software was used for data analysis. All data are expressed as mean ± standarddeviation. If there is a normal distribution of data, ANOVA was used followed by the Holm-Sidak method for data with homogeneity of variance or by Tamhane's T2 method for data without homogeneity of variance. If the data does not conform to normal distribution, the Wilcoxon rank-sum test was used. *P* < 0.05 was considered to be statistically significant.

## 3. Results

### 3.1. CEUS Assessment of Lesion Size and Blood Perfusion Changes in Nonluminal Breast Cancer after NAC Is Superior to Conventional Ultrasound

**Figure 1:**
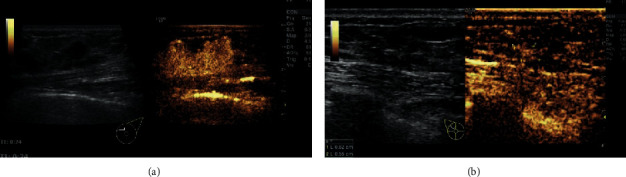
Contrast-enhanced ultrasound measurement of nonluminal breast cancer with nonconcentric shrink mode before and after NAC. (a) Left, two-dimensional ultrasound image of the lesion before NAC; right, ultrasound contrast image of the lesion before NAC; (b) Left, two-dimensional ultrasound image of the lesion after NAC; right, ultrasound contrast image of the lesion after NAC.

**Figure 2:**
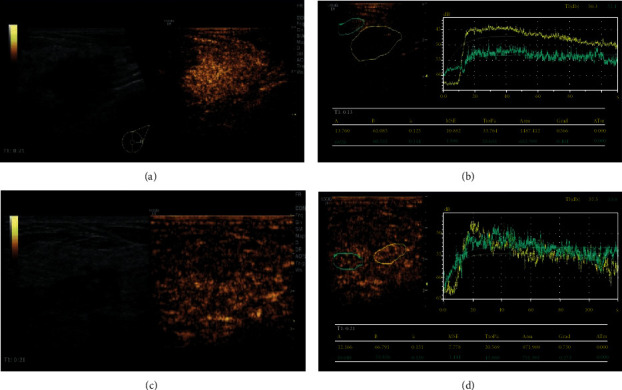
Contrast-enhanced ultrasound before and after NAC in nonluminal breast cancer patients. (a) Contrast-enhanced ultrasound image before NAC showing high enhancement. (b) Time-intensity curve before NAC showing fast-in and high-enhancement relative to adjacent cancer tissue. (c) Contrast-enhanced ultrasound image after NAC. The lesions shrank, showing slightly lower enhancement without enlargement. (d) Time-intensity curve after NAC, showing equal-in and the same enhancement as the adjacent tissues.

In order to determine which of conventional ultrasound and CEUS is more accurate to measure lesions before and after NAC, we conducted a comparative study using two methods.  Figure 1 showed the lesion measure before (Figure 1(a)) and after NAC (Figure 1(b)). Statistically, after NAC, the maximum diameter of the lesion measured by CEUS was smaller than that of conventional ultrasound (*P* < 0.05) and was not significantly different than pathological size (*P* > 0.05, Table 2). The representative CEUS images, including enhancement pattern and TIC curves, before and after NAC, were shown in Figure 2. Before NAC, the lesion showed high enhancement (Figure 2(a)), and the TIC showed fast-in (Figure 2(b)) relative to adjacent cancer tissues. After NAC, the CEUS of the lesion showed the characteristics of low enhancement and low perfusion (Figure 2(c)) and slow-in on TIC (Figure 2(d)). After NAC, the enhancement decreased from 57.44% to 37.92%, and the perfusion defect decreased from 44.44% to 21.25%. The area of the lesion with enlarged enhancement was selected as the region of interest. Quantitative analysis was then performed before and after NAC (Table 3). It was found that, after NAC, the PI decreased, the AUC decreased, and TTP was longer (*P* < 0.05). There was no difference in the rising slope, elimination slope, and the initial enhancement. These results indicate that CEUS assessment of lesion size and blood perfusion changes in nonluminal breast cancer after NAC is superior to conventional ultrasound.

### 3.2. Identification of CSCs in Nonluminal Breast Cancer

**Figure 3:**
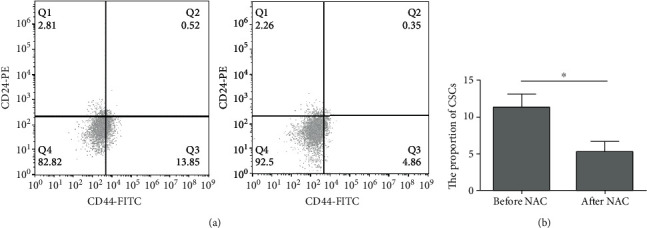
Analysis of the proportion of CSCs before and after NAC. Flow cytometry was performed. (a) Representative flow cytometry results. (b) Quantitative flow cytometry results. ^∗^*P* < 0.05.

In order to clarify the effect of NAC on CSCs in nonluminal breast cancer in the contrast-enhanced active area, we performed puncture sampling of the contrast-enhanced active area before NAC and after NAC. Then, CSCs were isolated in vitro. Flow cytometry was used to identify the isolated CSCs of breast cancer. The percentage of CD44^+^/CD24^-/^low cells was 5.283 ± 1.399% after NAC, significantly lower than that before NAC (11.289 ± 1.770%) (*T* = 10.311, *P* < 0.001, Figure 3). These results indicate that the number of CSCs in breast cancer is reduced after NAC.

### 3.3. Changes of CSC Marker and EMT Marker Expressions in Nonluminal Breast Cancer

**Figure 4:**
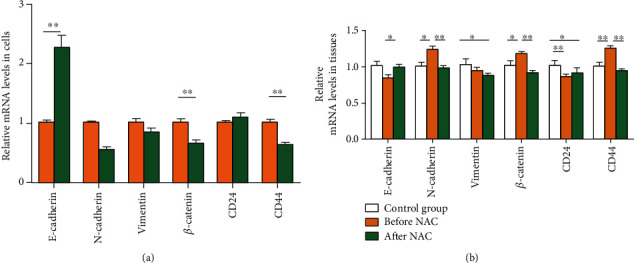
Relative mRNA expression of each gene in nonluminal breast cancer before and after NAC. (a) In breast tumor cells. (b) In tissue samples. ^∗^*P* < 0.05, ^∗∗^*P* < 0.01.

This study also examined the expression of CSCs markers (CD44 and CD24) and EMT markers (E-cadherin, *β*-catenin, vimentin, and N-cadherin) in the isolated breast tumor cells and in tumor tissues by qRT-PCR. The results showed that there was no significant change in *CD24* mRNA expression in breast tumor cells (*T* = −1.116, *P* = 0.278) (Figure 4(a)). The expression of *CD44* mRNA in breast tumor cells was significantly downregulated after NAC (*T* = 6.387, *P* < 0.001). Additionally, after NAC, the mRNA expression of *E-cadherin* was significantly upregulated (*T* = −6.255, *P* < 0.001); those of *N-cadherin* and *β-catenin* were significantly downregulated (*T* = 8.977 and 5.197, *P* < 0.001); that of *Vimentin* mRNA was not significantly changed (*T* = 1.920, *P* = 0.065). In tissues (Figure 4(b)), the mRNA expressions of *β-catenin*, *N-cadherin*, and *CD44* were significantly higher in the before NAC group than in the control group, and the expression of *CD24* was significantly lower in the before NAC group than in the control group. The mRNA expressions of *β-catenin*, *N-cadherin*, and *CD44* expression were significantly downregulated, and the expression of *E-cadherin* was significantly upregulated. The mRNA expression of *Vimentin* and *CD24* did not change significantly.

**Figure 5:**
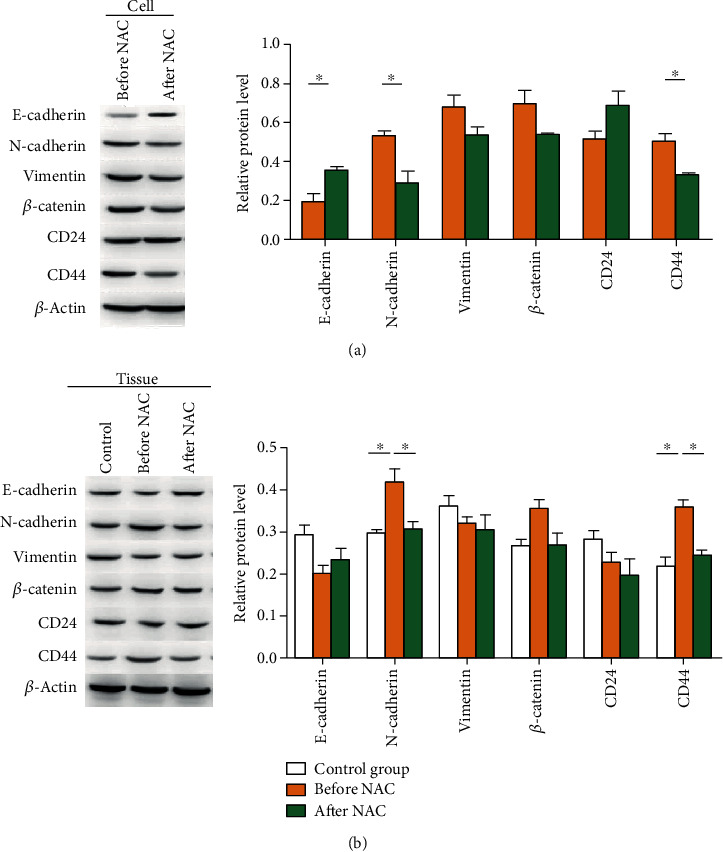
Detection of various indicators in non-luminal breast cancer primary cells and tissues before and after NAC. Western blot was performed to detect protein expression. (a) Representative and quantitative Western blot results in breast tumor cells. (b) Representative and quantitative Western blot results in tissues.

To further verify the above results, a Western blot was performed. As shown in Figure 5(a), in breast tumor cells, compared with before NAC, CD44 level was significantly downregulated after NAC (*T* = 4.374, *P* = 0.012), but there was no significant change in CD24 (*T* = −2.068, *P* = 0.108). After NAC, the expression of E-cadherin was significantly upregulated (*T* = −3.912, *P* = 0.017); that of N-cadherin was significantly downregulated (*T* = 3.775, *P* = 0.020); those of *β*-catenin and vimentin were downregulated insignificantly (*T* = 2.253, 1.979, *P* = 0.151, 0.119). As shown in Figure 5(b), in the cancer tissues before NAC, the levels of N-cadherin and CD44 were significantly higher than those in the control group (*P* < 0.05). However, no significant differences were found in *β*-catenin, E-cadherin, Vimentin, and CD24 between control and before NAC (*P* > 0.05). Compared with those before NAC, the expressions of N-cadherin and CD44 were significantly downregulated in cancer tissues after NAC (*P* < 0.05). However, there were no significant differences in E-cadherin, *β*-catenin, Vimentin, and CD24 between cancer tissues before and those after NAC (*P* > 0.05).

**Figure 6:**
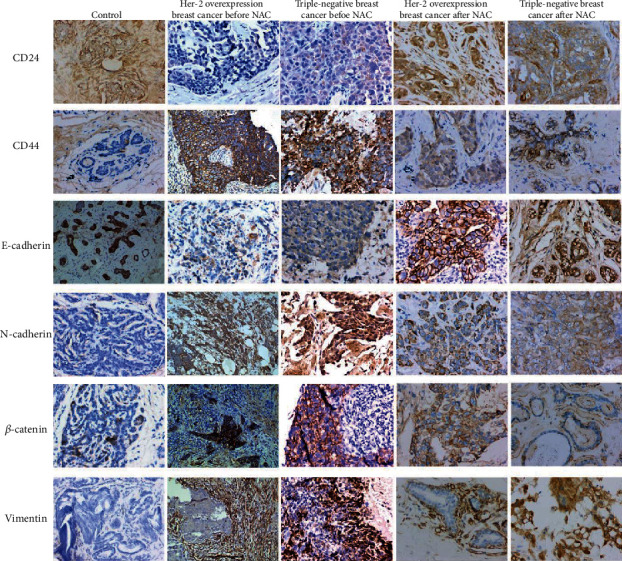
Immunohistochemical analysis of related proteins before and after NAC. Magnification: ×200. Immunohistochemistry was conducted to analyze CSC markers (CD44 and CD24), EMT markers (E-cadherin, *β*-catenin, vimentin, and N-cadherin) in benign breast lesions (control), and tumor tissues of Her-2 overexpression breast cancer and Her-2 overexpression breast cancer before and after NAC.

Moreover, immunohistochemistry was conducted to analyze CSCs markers (CD44 and CD24) and EMT markers (E-cadherin, *β*-catenin, vimentin, and N-cadherin) in benign breast lesions (control), paracancer tissues, and tumor tissues. The results showed that, compared with the control group, CD24, CD44, N-Cadherin, *β*-catenin, and Vimentin in tumor tissues before NAC were significantly overexpressed, and CD24, CD44, E-cadherin, N-cadherin, *β*-catenin, and Vimentin were significantly lower expressed in paracancer tissues before NAC (Figure 6 and Table 4). Compared with before NAC, the expressions of CD44, N-cadherin, *β*-catenin, and Vimentin in tumor tissues were significantly downregulated after NAC, while the expressions of E-cadherin and CD24 had no statistically significant change after NAC. Compared with the paracancer tissue before NAC, the expressions of CD44 and E-cadherin were significantly higher in the paracancer tissue after NAC, and the expression of other indicators had no statistically significant changes (Table 4 and Figure 6).

### 3.4. Focused Lesions Tend to Have Low Expression of CSCs and EMT Markers before Chemotherapy and High Contrast Perfusion

**Figure 7:**
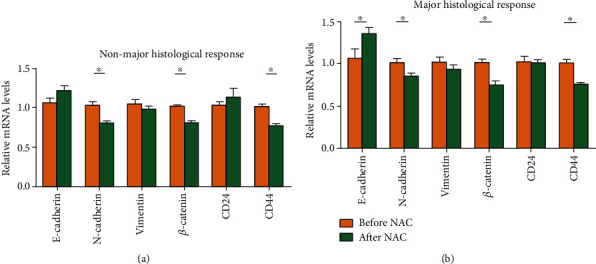
Different expressions of related genes with different NAC response. (a) Nonmajor histological response (NMHR). (b) Major histological response (MHR). ^∗^*P* < 0.05.

In order to evaluate the relationship among nonluminal breast cancer ultrasound contrast parameters, CSCs, EMT, and efficacy of chemotherapy before and after NAC, we divided the treatment response into 45 cases with MHR (56.25%) and 35 cases with NMHR (43.75%). The mRNA levels of CSCs markers (CD44 and CD24) and EMT markers (E-cadherin, *β*-catenin, vimentin, and N-cadherin) in tumor tissues were analyzed with qRT-PCR. The results showed that, in the NMHR group, the mRNA expressions of *CD44*, *N-cadherin*, and *β-catenin* were significantly downregulated after NAC than before NAC (*P* < 0.0001), but the mRNA expressions of *E-cadherin*, *Vimentin*, and *CD24* were not significantly changed (*P* > 0.05) (Figure 7(a)). In the MHR group, the mRNA expressions of *CD44*, *N-cadherin*, and *β-catenin* were significantly reduced after NAC than before NAC (*P* < 0.0001) (Figure 7(b)). The mRNA expressions of *E-cadherin* and *Vimentin* were upregulated (*P* < 0.05) after NAC. There was no significant change in the mRNA expression of *CD24* (*P* > 0.05).

**Figure 8:**
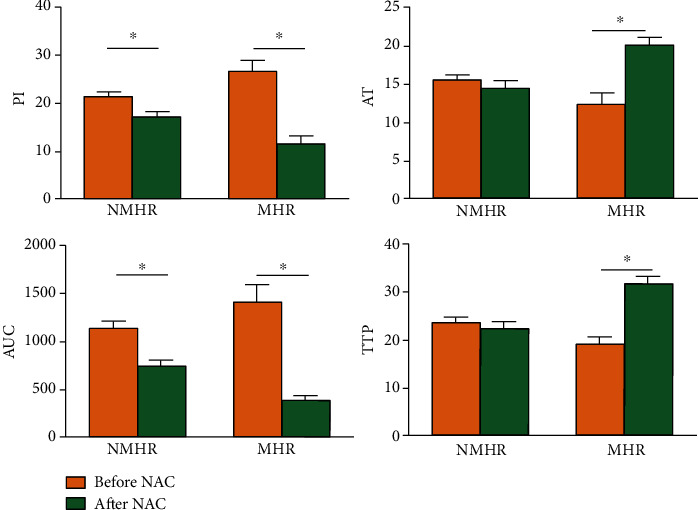
Differences in contrast-enhanced ultrasound parameters of nonluminal breast cancer with different effects before and after NAC. Parameters of PI (peak intensity), arrival time (AT), area under the curve (AUC), and time to peak (TTP) before and after NAC were analyzed between the MHR group and NMHR group. NMHR: Nonmajor histological response; MHR: major histological response. ^∗^*P* < 0.05.

The CEUS parameters before and after NAC were analyzed between the MHR group and NMHR group. For the NMHR group, the PI and AUC decreased after NAC than before NAC (*P* < 0.05), and the rest parameters remained not significantly changed (*P* > 0.05) (Figure 8). For the MHR group, all the CEUS parameters changed significantly after NAC. The PI decreased, the AT was longer, the AUC decreased, and TTP increased (*P* < 0.05).

Analysis of nonluminal breast cancer tissue samples before NAC showed that the mRNA expressions of *CD44* and *N-cadherin* in the NMHR group were higher than that of the MHR group (Table 5). The mRNA expressions of *E-cadherin* and *β-catenin* in the NMHR group were lower than that of the MHR group. For the CEUS parameters in the NMHR group, the PI was lower, and AT was late. This suggested that the nonluminal breast cancer of NMHR has a tendency of overexpression of CSC markers and EMT markers before the NAC relative to the MHR group, while the contrast-enhanced perfusion characteristics of the contrast group are slower and lower than the MHR group. This also means that nonluminal breast cancer with good efficacy tends to have low expression of CSC markers and EMT markers before NAC.

The cases with enlarged enhancement before NAC were 28.57% (10/35) in the NMHR group, which was lower than 55.56% (25/45) in the MHR group (*X*^2^ = 7.975, *P* = 0.005) before NAC. CEUS with perfusion defects was 20.00% (7/35) in the NMHR group, which was higher than 13.33% (6/45) in the MHR group (*X*^2^ = 9.212, *P* = 0.002). The number of patients with enhanced contrast enhancement after NAC was 54.28% (19/35) in the NMHR group, which was higher than 4.44% (2/45) in the MHR group (*X*^2^ = 7.975, *P* = 0.005). After NAC, contrast-enhanced patients with perfusion defect were 14.28% (5/35) in the NMHR group, which was higher than 11.11% (5/45) in the MHR group (*X*^2^ = 9.212, *P* = 0.002).

## 4. Discussion

Nonluminal breast cancer and luminal breast cancer may have different origins of CSCs. The former has a higher CSCs expression rate, and its stem cells have a stronger ability to divide symmetrically and to proliferate [17]. CEUS of nonluminal breast cancer also lacks typical malignant features, but it is superior in evaluating efficacy. Compared with enhanced MRI, CEUS can reflect tumor size more accurately and can display very small tumor blood vessels with a low flow rate of ≥100 *μ*m [18]. These blood vessels are abnormally enriched in the nonluminal breast cancer marginal zone [16]. This study suggests that the tumor size on CEUS was closer to that of postoperative pathology, but larger than that of two-dimensional ultrasound before NAC and significantly smaller than that of two-dimensional ultrasound after NAC. The extended range on CEUS than the two-dimensional ultrasound is considered the nonluminal breast cancer marginal zone (the enlarged enhancement area) [16]. Most scholars believe that CD44 + CD24-/low cells represent interstitial breast CSCs, which are mainly distributed in the edge of tumor invasion [19]. This study used CEUS to enlarge the enhanced area and focused on the most active area in nonluminal breast cancer lesions, which corresponds to the edge of tumor invasion.

As reported [20], it is difficult to evaluate the efficacy of NAC on nonluminal breast cancer. CEUS significantly reduces the degree of perfusion of the fibrosis tissue after NAC, which can truly show the size of the tumor and avoid underestimation of efficacy [13]. This study found no difference between CEUS measurements and pathological measurements in tumor diameter after NAC, but less than conventional ultrasound measurements, suggesting that nonluminal breast cancer does have nonconcentric shrink. This study confirms that CEUS is better than conventional ultrasound in estimating NAC efficacy on nonluminal breast cancer with nonconcentric shrink mode. Our results showed that the proportion of enhancement and perfusion defect was significantly reduced after NAC, and the microcirculation perfusion was slow-in and low enhancement. This change in blood perfusion is often earlier than the size of the lesion, so it is more objective to combine changes in lesion size and changes in microcirculation perfusion when evaluating the efficacy of NAC.

This study also found that the expressions of CSCs markers and EMT markers in breast tumor cells and tissues were significantly changed after NAC. Among them, CD44 was significantly downregulated, which is a most sensitive indicator, followed by the upregulation of E-cadherin and the downregulation of N-cadherin and *β*-catenin. *β*-Catenin is a key molecule of Wnt signaling and is the link of cell adhesion and proliferation during tumorigenesis and metastasis [21]. The downregulation of *β*-catenin also indicates the weakened cell proliferation after NAC [22]. Vimentin only showed decreased expression in the immunohistochemistry. There was no significant change in CD24. It has been shown that the expressions of CSC markers and EMT markers in nonluminal breast cancer after chemotherapy are increased, followed by reduced chemotherapy sensitivity of tumor cells [23]. However, the enrolled cases in this study underwent six cycles of NAC, which may eventually reduce the sensitivity of CSCs and EMT in nonluminal breast cancer to chemotherapeutic stress. As a result, the changes of CSCs and EMT marker proteins after chemotherapy in nonluminal breast cancer tissues are more complicated than in single-cell lines, which may present as downregulated expression of CSCs and EMT marker proteins in nonluminal breast cancer.

Furthermore, we found that E-cadherin expression was not significantly changed in nonluminal breast cancer with poor response to chemotherapy. In patients with good response to chemotherapy, E-cadherin was upregulated. E-cadherin can serve as a connective adhesive for epithelial cells, and its downregulation or nonexpression is the basis of epithelial cell dispersion [24]. Thus, the results of this study on E-cadherin indicate that nonluminal breast cancer, which responds well to chemotherapy, binds tightly to tumor epithelial cells after chemotherapy. Before NAC, the nonluminal breast cancer with poor response to chemotherapy showed relatively low expression of E-cadherin and *β*-catenin and relatively high expression of CD44 and N-cadherin compared to the MHR group. This indicates that the expression levels of CSCs and EMT markers in nonluminal breast cancer before chemotherapy is related to chemotherapy response, which is consistent with previous results [25]. In addition, in line with a previous study [26], nonluminal breast cancer lesions with poor response to chemotherapy were relatively hypoperfused but were accompanied by high expression of CSCs and EMT markers before NAC.

## 5. Conclusions

In summary, lesions with the characteristics of enlarged enhancement and perfusion defect on CEUS may have more CSCs and EMT. As a result, the prognosis may be poor. CEUS may predict the prognosis of luminal breast cancer before NAC. Our findings may help the early prediction of NAC efficacy and guide the individualized preoperative treatment.

## Figures and Tables

**Table 1:** Primers used in qRT-PCR analysis.

Primer name	Sequence (5′ to 3′)
E-cadherin-F	CGAGAGCTACACGTTCACGG
E-cadherin-R	GGGTGTCGAGGGAAAAATAGG
*β*-Catenin-F	AGCTTCCAGACACGCTATCAT
*β*-Catenin-R	CGGTACAACGAGCTGTTTCTAC
Vimentin-F	AGTCCACTGAGTACCGGAGAC
Vimentin-R	CATTTCACGCATCTGGCGTTC
N-cadherin-F	AGCCAACCTTAACTGAGGAGT
N-cadherin-R	GGCAAGTTGATTGGAGGGATG
CD44-F	CTGCCGCTTTGCAGGTGTA
CD44-R	CATTGTGGGCAAGGTGCTATT
CD24-F	CTCCTACCCACGCAGATTTATTC
CD24-R	AGAGTGAGACCACGAAGAGAC
hsa-Actin-F2	ACAGAGCCTCGCCTTTGCC
hsa-Actin-R2	GAGGATGCCTCTCTTGCTCTG

**Table 2:** Comparison of the maximum diameter of lesions before and after NAC by conventional ultrasound, contrast-enhanced ultrasound, and pathological measurement (mean ± standarddeviation).

Group	Case	Maximum diameter of lesions (mm)		
		Conventional ultrasound	CEUS	Pathological specimen
Before NAC	80	20.21 ± 7.03	28.45 ± 7.54^∗^	—
After NAC	80	14.23 ± 5.52	10.76 ± 3.34^∗^	11.56 ± 3.21^∗^

Notes: ^∗^*P* < 0.05, compared with conventional ultrasound.

**Table 3:** Changes in CEUS quantitative indicators before and after NAC in 80 nonluminal breast cancer patients (mean ± standarddeviation).

Group	Peak intensity (dB)	Area under the curve (dB/s)	Arrival time (s)	Rising slope (dB/s)	Elimination slope (dB/s)	Time to peak (s)	Initial enhancement (dB)
Before NAC	17.584 ± 5.762	1329.492 ± 352.493	15.333 ± 4.717	1.756 ± 0.655	0.850 ± 0.314	23.725 ± 8.042	−65.741 ± 4.330
After NAC	11.902 ± 4.503	875.441 ± 360.554	8.134 ± 2.327	1.556 ± 0.255	0.626 ± 0.340	26.864 ± 10.568	−62.504 ± 4.371
*t* value	3.122	3.936	3.409	2.016	1.607	2.208	0.866
*P* value	0.009	0.002	0.005	0.054	0.134	0.045	0.393

**Table 4:** Analysis of immunohistochemical data of various indicators in nonluminal breast cancer tissue (*n* = 80).

Groups	CD24	CD44	E-cadherin	N-cadherin	*β*-Catenin	Vimentin
Control	1.333 ± 1.047	1.533 ± 1.060	2.600 ± 1.242	0.200 ± 0.561	1.533 ± 1.125	1.933 ± 0.961
Precancer tissue before NAC	0.000 ± 0.000^△^	0.000 ± 0.000^△^	0.250 ± 0.500^△^	0.000 ± 0.000	0.000 ± 0.000^△^	3.250 ± 0.957^△^
Tumor tissue before NAC	2.259 ± 1.278^△^	2.931 ± 1.241^△^	2.948 ± 1.343	2.017 ± 1.235^△^	2.586 ± 1.200^△^	3.431 ± 0.797^△^
Precancer tissue after NAC	0.300 ± 0.675^△^	2.800 ± 0.789^△▲^	2.700 ± 0.823^▲^	0.000 ± 0.000	1.000 ± 1.155	2.100 ± 1.101
Tumor tissue after NAC	2.161 ± 1.604	2.143 ± 1.257^▽^	3.232 ± 0.953	1.107 ± 1.384^△▽^	1.393 ± 1.289^▽^	3.025 ± 1.113^△▽^

Note: ^△^: compared with the control group, *P* < 0.01; ^▲^: compared with paracancer tissue before NAC, *P* < 0.01; ^▽^: compared tumor tissue before NAC, *P* < 0.01. Because the data do not conform to the normal distribution, a rank-sum test was used for analysis.

**Table 5:** The relative mRNA expression of each index and CEUS parameters in nonluminal breast cancer tissue before NAC between groups with different pathological responses (mean ± standarddeviation, *n* = 80).

Group	mRNA level					CEUS parameters				
	*E-cadherin*	*β-Catenin*	*Vimentin*	*N-cadherin*	*CD44*	*CD24*	Peak intensity (dB)	Area under the curve (dB/s)	Arrival time (s)	Time to peak (s)
Nonmajor histological response	0.895 ± 0.066	0.920 ± 0.143	0.950 ± 0.274	1.334 ± 0.388	1.203 ± 0.066	0.845 ± 0.204	21.141 ± 7.744	15.488 ± 4.371	1123.600 ± 509.875	23.512 ± 7.788
Major histological response	1.156 ± 0.028	1.132 ± 0.167	0.943 ± 0.384	1.159 ± 0.199	1.032 ± 0.017	0.912 ± 0.309	26.612 ± 8.021	12.308 ± 5.376	1399.726 ± 616.251	19.077 ± 5.923
*T*/*Z* value	-3.912	-2.047	-0.830	-2.142	4.374	-0.223	-2.201	2.161	-1.618	1.883
*P* value	0.017	0.028	0.407	0.037	0.012	0.824	0.032	0.035	0.112	0.065

## Data Availability

Data of this study are available from the corresponding author on request.
